# Accelerated metabolic susceptibility to type 2 diabetes in older women with a history of gestational diabetes

**DOI:** 10.1530/EC-12-0072

**Published:** 2013-03-20

**Authors:** Alice S Ryan, John C McLenithan, Gretchen M Zietowski

**Affiliations:** 1 From the VA Maryland Health Care System, Research Service, Division of Gerontology and Geriatric Medicine, Department of Medicine Baltimore Veterans Affairs Medical Center 10 North Greene Street GRECC (BT/18/GR), Baltimore, Maryland, 21201 USA; 2 Division of Endocrinology, Diabetes and Obesity, Endocrinology, Diabetes and Nutrition at the University of Maryland School of Medicine 660 West Redwood Street HH 490, Baltimore, Maryland, 21201 USA; 3 The Baltimore Geriatric Research, Education and Clinical Center (GRECC) 10 North Greene Street GRECC (BT/18/GR) Baltimore VAMC, Baltimore, Maryland, 21201USA

**Keywords:** GDM, Glucose metabolism, Obesity, Postmenopausal women

## Abstract

The purpose of this study is to compare central obesity, insulin sensitivity, and cardiovascular disease risk factors between premenopausal and postmenopausal women with a history of gestational diabetes mellitus (GDM), controls, and women with type 2 diabetes (T2DM). Subjects were 73 overweight/obese and sedentary women who had a history of GDM (*n*=31) and were either premenopausal (*n*=11, 44±1 years, X±s.e.m.), postmenopausal (*n*=20, 58±1 years), or without a history of GDM as healthy postmenopausal controls (*n*=27, 57±1 years) or postmenopausal with T2DM (*n*=16, 59±1 years). The premenopausal GDM women had higher maximal oxygen uptake and lower visceral fat than the other three groups (*P*<0.05). BMI, %body fat, subcutaneous abdominal fat, and intramuscular fat did not differ significantly among the four groups. Glucose utilization (M, 3 h 40 mU/m^2^ per min hyperinsulinemic–euglycemic clamps) was 27% higher (*P*=0.05) in pre- than postmenopausal GDM and was not different between premenopausal GDM and postmenopausal controls. M was 28% lower (*P*=0.06) in postmenopausal GDM than controls and was not significantly different between postmenopausal GDM and T2DM groups. Thus, despite being younger and more physically fit, premenopausal women with prior GDM display similar central obesity, glucose, and metabolic profiles as postmenopausal controls. Postmenopausal women with prior GDM are more insulin resistant than controls of similar age, adiposity, and fitness levels and display comparable glucose utilization rates as similar as women with T2DM suggesting that a prior history of GDM may be an early manifestation of increased risk of later T2DM.

## Introduction

Women with a history of gestational diabetes mellitus (GDM) are at an increased risk for the development of type 2 diabetes mellitus (T2DM) within 5 years following pregnancy (1, 2) with the reported incidence of T2DM ranging from almost 3% to >70% in studies on women examined between 6 and 28 weeks *post partum* (3). Defects in glucose tolerance, insulin secretion, and insulin action are observed in women with a prior history of GDM (4, 5, 6, 7, 8). Likewise women with prior GDM compared with women without such a history have higher BMI, higher blood pressure, and worse lipid profiles (9, 10). Given these observations, as well as the fact that diabetes is a well-established cardiovascular disease (CVD) risk equivalent, women with prior GDM are at a heightened risk for subsequent CVD complications.

Aging is also associated with increased obesity, insulin resistance, and CVD risk (11, 12, 13). Thus, as women age with a history of GDM, they may be faced with a worsening of CVD risk factors and insulin resistance compared with their peers. Clinical observations, descriptive characteristics, and metabolic studies on women with a prior history of GDM, in general, are made within a 5-year *post partum* period. Thus little is known about lipoprotein and glucose metabolism in middle-aged and older woman with a prior history of GDM.

We hypothesized that pre- and postmenopausal women who have a history of GDM would be more obese with lower aerobic fitness, have greater visceral adiposity and be more insulin resistant than healthy postmenopausal controls with no history of GDM, and have comparable metabolic profiles to women with T2DM. Thus, this study compares insulin sensitivity and CVD risk factors in overweight, obese, sedentary women with and without a history of GDM, and never GDM women with women with T2DM. Insight into these abnormalities could help direct preventive treatments to decrease the risk for T2DM and CVD as women with a history of GDM age.

## Materials and methods

All subjects were recruited to be overweight and obese (BMI>25 kg/m^2^, range 25–40 kg/m^2^) women between the age of 38–65 years. Only women who were weight stable (<2.0 kg weight change in past year) and sedentary (<20 min of aerobic exercise 2×/week) were recruited. Subjects were screened by medical history questionnaire, physical examination, fasting blood profile and a graded exercise treadmill test in an attempt to exclude those with CVD. The women underwent a 2 h 75 g oral glucose tolerance test (OGTT) to classify diabetes status (14). Blood samples were only drawn at baseline and at the 2 h period for measurement of plasma glucose in women without a history of GDM. All subjects were nonsmokers, showed no evidence of cancer, liver, renal or hematological disease, or other medical disorders and were not on hormone replacement therapy. Women who were premenopausal had regular menstrual cycles and had not used hormonal contraception during the previous year. Postmenopausal women had not menstruated for at least 1 year and had plasma FSH levels >30 mIU/ml. Seventy-three women met all study criteria and were enrolled into the study. Of the 73 women, 31 pre- and postmenopausal women had a prior history of GDM (5–32 years ago), 26 postmenopausal women did not have a history of GDM (controls), and 16 postmenopausal women had T2DM without a history of GDM. Women who reported a history of GDM confirmed that the diagnosis was made by a physician or health care provider. The Institutional Review Board of the University of Maryland approved all methods and procedures for the study. Each participant provided written informed consent.

### Maximal oxygen uptake

Maximal oxygen uptake (VO_2_max) was measured using a continuous treadmill test protocol as previously described (11) with validation for attainment of VO_2_max. Seated blood pressures were determined twice before the exercise test and the average determination was used in the statistical analyses.

### Body composition

Height (cm) and weight (kg) were measured to calculate BMI as weight (kg)/height (m^2^). Waist circumference (at the narrowest point superior to the hip) was divided by the circumference of the hip (at the greatest gluteal protuberance) to obtain waist-to-hip ratio (WHR). Fat mass and lean tissue mass+bone mineral content=fat-free mass (FFM) were determined by dual-energy X-ray absorptiometry (Model DPX-L LUNAR Radiation Corp., Madison, WI, USA, version 1.3z analysis). A single 5 mm computed tomography scan was taken at the L_4_–L_5_ region using a PQ 6000 Scanner (General Electric Hi-Light, Cleveland, OH, USA) to quantitate visceral (VAT) and subcutaneous abdominal adipose tissue (SAT) areas and sagittal diameter (11). A second scan at the level of the mid-thigh was used to quantify muscle area, total fat area, and low-density lean tissue of the thigh (11).

### Metabolic testing

To control intake of nutrient before the metabolic studies, all subjects were provided with a eucaloric diet (50–55% carbohydrate, 15–20% protein, ≤30% fat) by a registered dietitian for 2 days before testing. The diet was composed of at least 150 g carbohydrate/day (15). The number of calories given to each woman was estimated from the 7-day food record and estimates of energy expenditure (16). All testing was performed in the morning after a 12 h overnight fast. All subjects were weight stabilized (<1 kg) for at least 2 weeks before metabolic testing. Metabolic testing was performed during the follicular phase of the menstrual cycle for the premenopausal women.

#### Lipoprotein lipids

The average of 2–3 fasting blood samples drawn on separate days was used in the determination of lipoprotein lipids. Blood samples were transferred into chilled tubes containing 1 mg of EDTA per cc of blood. Plasma was separated by centrifugation at 4 °C for 15 min at 2000 ***g***. Total cholesterol (TC), triglyceride (TG) concentrations, high-density-lipoprotein (HDL) cholesterol, and HDL_2_ cholesterol were measured as previously described (17) and low-density-lipoprotein cholesterol (LDL-C) was calculated by the Friedewald equation (18).

#### Hyperinsulinemic–euglycemic clamps

Peripheral tissue sensitivity to exogenous insulin was measured using a 3 h hyperinsulinemic–euglycemic clamp technique (19) with a priming and continuous infusion of insulin (240 pmol/m^2^ per min, Humulin, Eli Lilly Co.). Briefly, an i.v. catheter was inserted by percutaneous venipuncture for the infusion of glucose and insulin. A second catheter was inserted in a retrograde fashion into a dorsal hand or wrist vein, and the hand was enclosed in a grounded, insulated chamber, warmed to 70 °C to ‘arterialize’ (20) the blood obtained for all samples. For the assessment of basal glucose and insulin levels, three arterialized blood samples were drawn at 10-min intervals. Blood samples were obtained every 5 and 10 min thereafter for the determination of plasma glucose and insulin levels.

The mean plasma glucose level during 10–180 min of the euglycemic clamp was computed for each individual study and expressed as a percentage of the desired goal. This was 97.7±0.4, 97.1±0.3, 97.7±0.2, and 97.1±0.5% of the desired goal with a CV of 4.7±0.7, 4.5±0.6, 4.7±0.3, 5.2±0.5% for the groups respectively.

#### Indirect calorimetry

Continuous indirect calorimetry was performed before the start of the glucose infusion and during the last 30 min. of the insulin infusion by the open circuit dilution technique using a SensorMedics DeltaTrac cart (Yorba Linda, CA, USA). Rates of glucose oxidation were calculated from measurements of CO_2_ production and O_2_ consumption using established equation (21) with correction for protein oxidation determined from 24 h urinary urea nitrogen. Nonoxidative glucose metabolism was calculated as the difference between total glucose uptake and glucose oxidation.

#### Analysis of blood samples

Blood samples were collected in heparinized syringes and placed in prechilled test tubes containing 1.5 mg EDTA/ml of blood in a total volume that was 4% of the sample volume. The blood samples were centrifuged at 4 °C and a 1 ml aliquot of plasma was rapidly frozen (80 °C) for subsequent hormone analysis. Determinations were performed in duplicate. Plasma glucose was measured with the glucose oxidase method (Beckman Instruments, Fullerton, CA, USA) and plasma insulin by RIA (Millipore, St Charles, MO, USA).

### Statistical analyses

Glucose utilization (M) for 30 min intervals was calculated as described (22). Statistical significance among groups was determined by one-way ANOVA and *post-hoc* LSD testing. Relationships between variables were determined by linear regression analyses and calculation of Pearson correlation coefficients. All data were analyzed by SPSS statistical software (SPSS, Inc.). Data are expressed as mean±s.e.m. and significance was set at the *P*<0.05 level.

## Results

The physical characteristics of the women are presented in Table 1. The GDM history group (*n*=31) was divided into two groups by menopausal status and age (<50 years, *n*=11 and ≥50 years, *n*=20) and compared with postmenopausal controls (*n*=26) and T2DM (*n*=16). By design, the premenopausal GDM women were younger (*P*<0.01) than the three postmenopausal groups that were of similar age. The premenopausal GDM women had between a 15 and 23% higher VO_2_max (ml/kg per min) and 38 and 43% lower VAT than each of the other three groups (*P*<0.05). BMI, % body fat, fat mass, and FFM did not differ significantly among the four groups. There were also no differences in waist circumference, WHR, subcutaneous abdominal fat, sagittal diameter, and mid-thigh low-density lean tissue (intramuscular fat) between any of the groups. Mid-thigh muscle area was 19% higher in premenopausal GDM than controls (*P*<0.05) and 24% higher in premenopausal women than T2DM (*P*<0.01) but did not differ between pre- and postmenopausal GDM.

The blood pressure and lipoprotein lipids are reported in Table 2. Systolic (SBP) and diastolic blood pressures (DBP) were similar among the four groups. In addition, TC, LDL-C, HDL-C, HDL_2_-C, and TG levels did not differ between premenopausal GDM, postmenopausal GDM, controls, and T2DM. These results remained the same by excluding four women on TG lowering medications and nine women on hypertension (HTN) medications.

As a result of the screening OGTT in the 31 women with history of GDM, 14 women had normal glucose tolerance (NGT), nine had impaired glucose tolerance (IGT), and eight women had T2DM (one woman was on Glyburide treatment and the remaining seven women were untreated). The specific breakdown by groups is reported in Table 1. To examine the effects of age and menopause on glucose metabolism in women with the history of GDM, comparisons were performed between pre- and postmenopausal women with a history of GDM (Table 3). There were no differences in fasting glucose, fasting insulin, and G_120 min of OGTT_ between the two groups with a history of GDM. Insulin_120 min of OGTT_ was 49% higher in postmenopausal than premenopausal GDM (*P*<0.05). Glucose utilization, expressed in μmol/kg_FFM_ per min (M) was 27% higher in pre- than postmenopausal women with a history of GDM (*P*=0.05).

To examine the effects of a prior history of GDM on glucose metabolism, women with a history of GDM were compared with controls. Seventeen women in the control group had NGT and nine women had IGT. Fasting glucose and insulin levels were not different between postmenopausal women with a history of GDM and postmenopausal controls. However G_120_ was 27% higher (*P*<0.01), insulin_120_ was 33% higher (*P*<0.05), and M was 28% lower (*P*=0.06) in postmenopausal women with GDM history than postmenopausal controls. Oxidative glucose disposal was lower in the postmenopausal GDM group than controls (*P*<0.001). Furthermore, fasting glucose, fasting insulin, G_120_, insulin_120_, and M were not significantly different between premenopausal women with GDM history and postmenopausal controls.

Differences in glucose metabolism were next examined between similarly obese women with a history of GDM and women with T2DM and never GDM. None of the 16 T2DM women were on insulin, three women were on oral hypoglycemic agents (1 Glucotrol, 2 Glucophage), and the remaining 12 were not on medications and had undiagnosed diabetes before study entry. Both fasting glucose and G_120_ were higher in T2DM than pre- and postmenopausal women with a history of GDM (*P*<0.01). Fasting insulin and M were not significantly different between postmenopausal GDM and T2DM. Fasting insulin was lower, M was higher, and oxidative glucose disposal was higher in premenopausal women with GDM history than T2DM women (*P*<0.05).

Glucose utilization (μmol/kg per min) during the last 30 min of the hyperinsulinemic–euglycemic clamp in the GDM history group was negatively correlated with age (*r*=−0.41, *P*=0.05), WHR (*r*=−0.54, *P*=0.01), fat mass (*r*=−0.42, *P*<0.05), %fat (*r*=−0.44, *P*<0.05), trunk fat mass (*r*=−0.57, *P*<0.01), SAT (*r*=−0.54, *P*=0.01), sagittal diameter (*r*=−0.60, *P*<0.01), fasting insulin (*r*=−0.58, *P*<0.01), G_120_ (*r*=−0.51, *P*<0.05), and Insulin_120_ (*r*=−0.55, *P*<0.05). In a stepwise regression model with these variables, Insulin_120_ and WHR were independent predictors of M (cumulative *r*=0.67, *P*<0.005).

## Conclusions

To test our hypothesis of evaluating alterations in carbohydrate metabolism due to a history of GDM, we studied pre- and postmenopausal women with a history of GDM compared with postmenopausal controls and women with T2DM. Our results indicate that postmenopausal women with prior GDM are more insulin resistant than controls of similar age, adiposity, and fitness levels, and display comparable glucose usage rates as similarly characterized women with T2DM. Moreover, despite being younger, having lower VAT and higher aerobic capacity, premenopausal GDM women are comparable with postmenopausal control women in terms of glucose usage.

Several studies indicate differences in glucose metabolism between young GDM women and controls (7, 8, 23, 24), but there is no information on glucose metabolism of postmenopausal woman with a history of GDM. In a study on lean and obese prior GDM women more than 2 months after their index (most recent) pregnancy, insulin sensitivity index was lower in obese but not lean women with prior GDM compared with controls (7, 8). Normal-weight glucose tolerant young women with prior GDM had significantly decreased glucose usage compared with controls (23). African-American women with a history of GDM and parental history of T2DM had defects in β-cell secretion and lower insulin sensitivity than healthy controls of similar age and obesity (24), suggesting that a positive history of GDM adds further to the metabolic risk than parental history of diabetes alone. Our results compliment these findings by showing that postmenopausal women with a history of GDM have comparable glucose usage rates as similar as phenotypic women with T2DM and that younger, premenopausal, more physically fit women with prior GDM have similar insulin sensitivity to older, postmenopausal sedentary controls. Our results suggest that a prior history of GDM has detrimental effects on glucose homeostasis even later in life in postmenopausal women.

Central distribution of adiposity and intramuscular fat are associated with IGT, T2DM, and insulin resistance (11, 12, 13). One study reports that obese women with prior GDM soon after delivery had higher WHR than matched controls (7, 8). We are unaware of any studies that have measured visceral or subcutaneous abdominal fat in postmenopausal women with prior GDM. Two studies have examined visceral fat in younger women (mean age ∼32 years) with a history of GDM (25, 26). In GDM women studied, three months *post partum*, visceral fat and subcutaneous fat were similar in women who breastfed and those who did not (25), but there was no control group. In contrast, visceral fat was greater in Asian women with a history of GDM with IGT than age and BMI-matched GDM women with NGT and normal controls (27). A study reports increased intramyocellular lipid (soleus and tibialis-anterior muscles) in young women ∼6 months after delivery with GDM (17). Our results indicate that younger GDM women have less VAT than postmenopausal women with and without a history of GDM. Furthermore there were no differences in VAT between the postmenopausal controls and GDM groups, suggesting that aging and/or menopause and not a history of GDM *per se* contributes to the increased deposition of VAT in the older women. Moreover, although younger women with a history of GDM had greater muscle areas of the mid-thigh, levels of intramuscular fat were similar to each of the older groups. As liver fat content is associated with insulin resistance in women with a history of GDM that is independent of obesity (26), it is possible that liver fat contributed to reduced insulin sensitivity in older women with a history of GDM. Our regression analyses in women with a history of GDM indicate that total body fat mass and subcutaneous abdominal fat are associated with insulin resistance, demonstrating the importance of increased body fat and central obesity to increase the risk for the development of T2DM years after pregnancy complicated by GDM.

Women with prior GDM compared with women without such a history have a worse lipid profile including an increased TC, LDL-cholesterol, and TG levels (9). Moreover CVD risk factors (BMI, hypertension, and abnormal lipids) are higher at the *post partum* visit in women with prior GDM with impaired fasting glucose than prior GDM women with normal glucose status, suggesting that a continuation of abnormal glucose metabolism following delivery is associated with other CVD risk factors (28). TG levels were increased and HDL levels were decreased 3–11 months *post partum*, but lipid abnormalities disappeared after this point and were not different than controls up to 3 years after delivery (29). In our study on women at least 5 years *post partum*, we found no difference in lipoprotein lipid profiles or SBP and DBP between controls and prior GDM women, suggesting that the length of follow-up and degree of obesity may also influence whether lipoprotein levels are altered in women with prior GDM.

As this is a cross-sectional study, we cannot analyze within each woman how the menopause transition modifies the metabolic characteristics. However, we are able to suggest that within women with a history of GDM, menopause likely contributed to a worsening of insulin sensitivity. Limitations of this clinical investigation are the small sample size and lack of control group for the premenopausal women with GDM. However our previous work indicates that normal weight premenopausal women without a history of GDM have similar VO_2_max and visceral fat (30) and higher M (22). The literature concerning glucose metabolism in prior GDM women is limited to women who were studied in their mid-thirties, ∼15 years younger than our cohort, with follow-ups of <5–6 years *post partum*, considerably shorter than ours. Yet, it is the older, postmenopausal women with a history of GDM who are at high risk to develop T2DM. Additional strengths of our study include direct measures of insulin sensitivity using the glucose clamp (with insulin levels that suppress hepatic glucose production), diet control in that subjects were given prepared meals, and control of physical activity level before the metabolic studies; thus controlling for known factors that could alter glucose metabolism, lipid, and blood pressure levels. Future studies could address skeletal muscle metabolism including fat oxidation and mitochondrial function, inflammatory markers, or genetic analyses as areas to explain the increased insulin resistance in older women with a history of GDM.

In summary, total and central obesity, physical fitness, CVD risk factors, and glucose usage were evaluated in overweight and obese women with prior GDM and were compared with control women or women with T2DM. We found that a history of GDM is associated with insulin resistance even in women many years after gestation complicated by GDM. Thus, older overweight and sedentary women with a history of GDM may require treatment and would benefit from diet-induced weight loss or an exercise program to enhance insulin sensitivity and prevent progression to T2DM.

## Figures and Tables

**Table 1:** Physical characteristics, total and regional body composition of overweight, sedentary women with a history of GDM, controls, and women with type 2 diabetes.

	**Premenopausal GDM** (*n*=11)	**Postmenopausal GDM** (*n*=20)	**Postmenopausal controls** (*n*=26)	**Postmenopausal T2DM** (*n*=16)
Age (years)	44±1*^,^^†^^,^^‡^	58±1	57±1	59±2
Race (AA/C)	0/11	6/14	5/21	2/14
NGT/IGT/T2DM	6/3/2	8/6/6	17/9/0	0/0/16
Weight (kg)	80.9±3.3	86.2±2.1	87.7±3.0	85.8±4.2
BMI (kg/m^2^)	31.4±1.2	33.3±0.8	33.2±1.0	33.6±1.4
Waist circumference (cm)	91.0±1.9	96.8±2.1	95.7±2.2	98.3±3.8
Waist-to-hip ratio	0.81±0.01	0.83±0.01	0.82±0.01	0.85±0.02
VO_2_max (ml/kg per min)	23.3±1.6*^,^^†^^,^^‡^	20.2±0.9	19.8±0.5	18.0±1.2
Percent body fat	41.6±2.2	46.3±0.9	46.1±1.0	45.8±1.8
Fat mass (kg)	33.8±3.2	39.7±1.5	39.7±2.0	39.8±2.8
Fat-free mass (kg)	45.7±0.8	47.8±1.8	45.6±1.4	50.4±3.4
VAT (cm^2^)	101.0±8.9*^,^^†^^,^^‡^	169.5±12.5	163.0±12.1	176.6±24.1
SAT (cm^2^)	393.9±44.2	467.7±22.0	482.4±26.2	450.7±31.9
Sagittal diameter (cm)	23.6±0.8	26.9±0.7	26.6±0.6	26.2±1.0
Mid-thigh fat (cm^2^)	166.3±19.6	189.8±14.9	197.8±14.1	141.6±13.5^§^^,^^∥^
Mid-thigh muscle (cm^2^)	94.0±3.8^†^^,^^‡^	81.0±4.9	79.0±3.0	75.2±4.5
Mid-thigh low density lean tissue (cm^2^)	15.4±1.6	18.4±2.9	17.9±2.2	16.7±2.3

VAT, visceral adipose tissue area; SAT, subcutaneous abdominal adipose tissue area. Values are means±s.e.m. *P<0.05 premenopausal GDM vs postmenopausal GDM. ^†^ *P*<0.05 premenopausal GDM vs controls. ^‡^ *P*<0.05 premenopausal GDM vs postmenopausal T2DM. ^§^ *P*<0.05 postmenopausal GDM vs postmenopausal T2DM. ^∥^ *P*<0.05 postmenopausal controls vs postmenopausal T2DM.

**Table 2:** Blood pressure and lipoprotein lipids in overweight, sedentary women with a history of GDM, controls, and women with type 2 diabetes.

	**Premenopausal GDM** (*n*=11)	**Postmenopausal GDM** (*n*=20)	**Postmenopausal controls** (*n*=26)	**Postmenopausal T2DM** (*n*=16)
Systolic blood pressure (mmHg)	123±5	129±4	134±3	139±4
Diastolic blood pressure (mmHg)	80±4	77±2	82±2	79±3
Total cholesterol (mmol/l)	4.96±0.21	5.31±0.24	5.14±0.16	4.74±0.31
LDL-cholesterol (mmol/l)	2.85±0.29	3.47±0.20	3.28±0.15	2.78±0.28
HDL-cholesterol (mmol/l)	1.33±0.14	1.20±0.06	1.25±0.06	1.24±0.08
HDL_2_-cholesterol (mmol/l)	0.30±0.09	0.17±0.03	0.15±0.04	0.16±0.04
Triglycerides (mmol/l)	1.69±0.47	1.41±0.13	1.30±0.11	1.52±0.16

Values are means±s.e.m.

**Table 3:** Insulin sensitivity in overweight, sedentary women with a history of GDM, controls, and women with type 2 diabetes.

	**Premenopausal GDM** (*n*=11)	**Postmenopausal GDM** (*n*=20)	**Postmenopausal controls** (*n*=26)	**Postmenopausal T2DM** (*n*=16)
Fasting plasma glucose (mmol/l)	5.5±0.2	5.9±0.2	5.5±0.1	7.1±0.4^‡^^,^^§^^,^^∥^
Fasting plasma insulin (pmol/l)	70±15	90±11	71±5	120±27^‡^^,^^∥^
Glucose_at 120 min of OGTT_ (mmol/l)	8.3±0.9	9.2±0.7^†^	6.7±0.4	13.4±0.6^‡^^,^^§^^,^^∥^
Insulin_at 120 min of OGTT_ (pmol/l)	386±86*	759±149^†^	504±14	668±264
10–180 min of clamp: glucose (mmol/l)	5.33±0.16	5.73±0.13	5.38±0.09	6.04±0.43
Insulin (pmol/l)	454±24	585±30	492±13	518±38
Last 30 min of clamp: (μmol/kg_FFM_ per min) glucose usage (M)	53.9±5.8*	39.2±5.8	50.3±3.2	32.7±6.5^‡^^,^^∥^
Nonoxidative glucose disposal	37.5±5.6	28.1±3.7	27.7±2.3	24.8±6.8
Oxidative glucose disposal	17.9±2.6	12.8±1.8^†^	22.2±1.6	9.8±1.7^‡^^,^^∥^

Values are means±s.e.m. OGTT, oral glucose tolerance test. *P≤0.05 premenopausal GDM vs postmenopausal GDM. ^†^ *P*<0.05 postmenopausal GDM vs postmenopausal controls. ^‡^ *P*<0.05 premenopausal GDM vs postmenopausal T2DM. ^§^ *P*<0.05 postmenopausal GDM vs postmenopausal T2DM. ^∥^ *P*<0.05 postmenopausal controls vs postmenopausal T2DM.
